# Phenolic Bioactives From Plant-Based Foods for Glycemic Control

**DOI:** 10.3389/fendo.2021.727503

**Published:** 2022-01-18

**Authors:** Dipayan Sarkar, Ashish Christopher, Kalidas Shetty

**Affiliations:** Department of Plant Science, North Dakota State University, Fargo, ND, United States

**Keywords:** alpha-amylase, alpha-glucosidase, antioxidant, anti-hyperglycemia, bioprocessing strategies, carbohydrate metabolism, phenolics, plant foods

## Abstract

Plant-based foods containing phenolic bioactives have human health protective functions relevant for combating diet and lifestyle-influenced chronic diseases, including type 2 diabetes (T2D). The molecular structural features of dietary phenolic bioactives allow antioxidant functions relevant for countering chronic oxidative stress-induced metabolic breakdown commonly associated with T2D. In addition to antioxidant properties, phenolic bioactives of diverse plant foods have therapeutic functional activities such as improving insulin sensitivity, reducing hepatic glucose output, inhibiting activity of key carbohydrate digestive enzymes, and modulating absorption of glucose in the bloodstream, thereby subsequently improving post-prandial glycemic control. These therapeutic functional properties have direct implications and benefits in the dietary management of T2D. Therefore, plant-based foods that are rich in phenolic bioactives are excellent dietary sources of therapeutic targets to improve overall glycemic control by managing chronic hyperglycemia and chronic oxidative stress, which are major contributing factors to T2D pathogenesis. However, in studies with diverse array of plant-based foods, concentration and composition of phenolic bioactives and their glycemic control relevant bioactivity can vary widely between different plant species, plant parts, and among different varieties/genotypes due to the different environmental and growing conditions, post-harvest storage, and food processing steps. This has allowed advances in innovative strategies to screen and optimize whole and processed plant derived foods and their ingredients based on their phenolic bioactive linked antioxidant and anti-hyperglycemic properties for their effective integration into T2D focused dietary solutions. In this review, different pre-harvest and post-harvest strategies and factors that influence phenolic bioactive-linked antioxidant and anti-hyperglycemic properties in diverse plant derived foods and derivation of extracts with therapeutic potential are highlighted and discussed. Additionally, novel bioprocessing strategies to enhance bioavailability and bioactivity of phenolics in plant-derived foods targeting optimum glycemic control and associated T2D therapeutic benefits are also advanced.

## Type 2 Diabetes Etiology: Role of Chronic Oxidative Stress, Hyperglycemia and Gut Health

Growing prevalence of diet and lifestyle-influenced non-communicable chronic diseases (NCDs) is severely affecting communities leading to breakdown in public health solutions across the globe. Since the beginning of 21^st^ century, NCD-associated morbidity and mortality has increased significantly contributing to emerging global public health crisis and health disparities (1). Due to its etiological link to sustained and accumulated unhealthy diets and lifestyle, poor urban communities living on subsidized and hyper-processed foods (calorie dense and nutritionally imbalanced) have become more vulnerable and susceptible to NCDs (2). This public health challenge has been exposed and has worsened with COVID-19 pandemic, as poor urban and suburban communities with underlying NCD related health conditions are being the most susceptible and has affected population globally (3, 4). Among common NCDs, type 2 diabetes (T2D) is one of the major contributors for poor public health outcomes and widening health disparities worldwide irrespective of current crisis with COVID-19 pandemic.

In 2019, there were 463 million people or 9.3% of the world’s population living with T2D and now estimated that 700 million or 10.9% will have the disease by 2045 (5). There are additional populations, who are undiagnosed or at pre-diabetic stage, making this chronic disease epidemic even more severe than what the current statistical estimation reflect. In addition to the higher prevalence and mortality rates associated with T2D, there is also the burden of economic stress due to health expenditure involved in the treatment of T2D and its associated complications. Between 2015 and 2019, the global expenditure on T2D was estimated to be around 673 - 760 billion USD (6, 7). Therefore, the complexity of T2D and its burden towards society across continents and countries is multifaceted, affecting all spheres of human life and wider community well-being. To find solutions towards this public health challenge and for designing well-rounded preventative strategies and interventions, it is important to understand the diet-linked metabolic breakdowns and common risk factors associated with pathogenesis and etiology of T2D.

Type 2 diabetes is largely a diet-linked metabolic disorder that is associated with insulin resistance, β cell dysfunction, abnormal glucose and lipid metabolism, and chronic oxidative stress-induced inflammation (8). Altered carbohydrate absorption, depletion of stored glycogen, increased gluconeogenesis, β cell dysfunction, insulin resistance in peripheral tissues, and altered insulin signaling pathways resulting from poor diet, associated lifestyle influenced metabolic breakdowns coupled with genetic factors, can contribute towards the risks of developing chronic hyperglycemia (8). These risk factors can potentially cause epigenetic changes *via* histone modification mechanisms such as DNA methylation, which in turn can impair the action and secretion of insulin thereby contributing towards chronic hyperglycemia and progression of T2D (9). A review of numerous studies has indicated that differential DNA methylation and gene expression can occur in muscle, adipose, liver, and pancreatic islets in individuals with T2D, when compared to non-diabetic individuals (9). Histone modification *via* DNA methylation of the PDX1 gene, which codes for a transcription factor involved in pancreatic development and β cell maturation, can lead to lower expression of this gene thereby contributing towards the pathogenesis of T2D, and gestational diabetes (9, 10).

Like chronic hyperglycemia, chronic oxidative stress resulting from cellular oxygen malfunction can also play a key role in the pathogenesis of T2D and its associated complications. Breakdown in cellular redox homeostasis under hyperglycemic conditions can lead to the generation of excessive reactive oxygen species (ROS) which impedes the normal functioning of cells (pancreatic β cells) and tissues (liver, adipose, and muscle) (11, 12). Therefore, cellular and tissue impairment due to oxidative stress represents an underlying mechanism of glucose toxicity in T2D and its related micro- and macro-vascular complications (13). Significantly higher levels of oxidative stress markers (malondialdehyde and catalase) and inflammatory markers (TNF-α and IL1-α) were observed in blood samples of patients affected with T2D, indicating that oxidative stress and inflammation can be triggered in the pathogenesis of diabetes (14). In recent times, the role of impaired mitochondrial function in the pathogenesis of T2D is gaining increasing relevance through various *in vivo* and *ex vivo* metabolic studies that have highlighted mitochondrial dysfunction as a predisposing condition for ectopic lipid deposition and insulin resistance (15). However, further clinical trials are needed to determine if the measurement of oxidative stress markers can be useful in the detection and can be integrated in therapeutic interventions against T2D (16).

Additionally, cellular and circulatory glucose and redox homeostasis critical for preventing and managing T2D are also associated with improved human gut health. Therefore, examining the role of gut microbiota in the pathogenesis T2D also has merit. The human gut microbiota consists of 6 major phyla which are Firmicutes, Bacteroidetes, Actinobacteria, Proteobacteria, Fusobacteria, and Verrucomicrobia, of which Firmicutes and Bacteroidetes are the dominant phyla that account for 90% of the gut microbiome (17). Studies have shown that loss of microbial diversity or dysbiosis can act as a factor in the rapid progression of insulin resistance in T2D (18). Dysbiosis can result in increased gut permeability, which allows for translocation of microbial pathogens and their products (e.g., lipopolysaccharides) into the blood stream circulation leading to metabolic endotoxemia, low grade inflammation, and insulin resistance, thereby contributing towards the pathogenesis of T2D (19, 20). Results from over 42 human studies including preclinical studies or clinical trials have shown that the genera of *Bifidobacterium*, *Bacteroides*, *Faecalibacterium*, *Akkermansia* and *Roseburia* were inversely associated with development of T2D through improved metabolism and gut health, while the genera of *Ruminococcus*, *Fusobacterium*, and *Blautia* contributed to T2D (20). However, more clinical, and epidemiological studies are needed to examine the specific role of gut microbiota in regulating carbohydrate metabolism and for improving overall glycemic control.

The current understanding on etiology and pathogenesis of T2D clearly indicates that maintaining cellular and circulatory glucose, redox homeostasis, and improving gut health are critical for countering chronic oxidative stress and hyperglycemia. Healthy diet coupled with regular exercise can help to maintain optimum metabolic regulation and functions, especially to support favorable cellular conditions for oxygen and nutrient coupling during energy (ATP) synthesis (oxidative phosphorylation) in the mitochondria (16). Therefore, the major aim of this review is to examine the role of plant food sources from different ecological origins with high health protective bioactive metabolites, specifically anti-diabetic functional role of phenolics, for countering chronic oxidative stress, chronic hyperglycemia, and for improving human digestive health, which are essential preventative measures to reduce the risks of developing T2D.

## Phenolic Bioactives of Plant Foods: Role in Human Metabolism and Glycemic Control

Nutritionally balanced and healthy diet has a major role in the prevention and management of T2D, and several studies suggest that greater consumption of whole grains, legumes, vegetables, and fruits with balanced nutritional profile and health protective bioactive compounds can potentially decrease the risk of T2D (21, 22). The current evidence clearly indicates that type of diet, dietary composition, and diversity of plant foods that are rich in micro-nutrients, dietary fiber, vitamins, and health protective bioactive compounds like dietary antioxidants are potentially beneficial to improve glycemic control and help counter T2D associated complications (23). However, understanding and optimizing different food sources and processing variables is essential to effectively integrate them into dietary and therapeutic interventions targeting T2D prevention and its management. Such T2D benefits targeted optimization can be achieved through identification of key nutritional biomarkers and functional bioactive components of plant foods that have the potential to modulate carbohydrate metabolism which are beneficial in maintaining cellular glucose homeostasis, redox homeostasis, and for improving human gut health. Plant-based foods are good sources of many bioactive compounds including phenolics, carotenoids, vitamins, alkaloids, saponins, and polysaccharides with diverse human health protective bioactivities (24). However, stress inducible phenolic bioactives of plant food sources are gaining wider attention in dietary and therapeutic interventions due to their specific role in countering chronic oxidative stress, hyperglycemia, dyslipidemia and improving digestive health, which are relevant for preventing T2D progression and pathogenesis (25). Therefore, examining the specific glycemic control relevant and health protective functional role of phenolic bioactives of plant foods from different ecological origins and their subsequent optimization based on sound metabolic rationale for dietary and therapeutic applications has significant merit.

Phenolic metabolites are widely distributed across and within all plant families (23). Therefore, improving diversity of plant foods also improves the diversity of these protective phenolic bioactive compounds in our daily diet (26). Such improvements in intake of plant foods enriched in health protective phenolics are relevant in reducing risks of T2D. However, composition and content of these phenolic bioactives vary widely among different plant foods and their bioavailability and functionality changes with different pre and post-harvest conditions (23, 27, 28).

Overall, phenolic bioactives of plant foods can be broadly classified into two major groups, flavonoid, and non-flavonoid compounds (29). When consumed through plant-based diet, these phenolic bioactives are partially absorbed in the stomach and small intestine while rest of the phenolics are utilized by microflora in the large intestine for microbial growth (prebiotic effect) and/or are converted into other phenolic derivatives through microbial action, followed by their absorption in the large intestine (30). It is estimated that 90–95% of consumed phenolics are not absorbed in the small intestine, but accumulate in the large intestinal lumen, where the gut microbiota have the metabolic capacity to convert the phenolic compounds into active metabolites, which in turn are responsible for the human health protective functions (31).

Following absorption in the stomach and small intestine, phenolic compounds and their derivatives are transported to the liver *via* enterohepatic circulation, where they undergo biotransformation and the resulting metabolites are transported *via* the bloodstream to other tissues and organs including pancreas, muscle, liver, and adipose tissue and/or are excreted from the body *via* urine (30). The rate or extent of absorption of phenolics is influenced by several factors such as the chemical structure of the compound as well as its gastrointestinal digestion, absorption, metabolic processing, and degree of conjugation (8, 32). The bioavailability and absorption of phenolics dictate their human health protective functionalities including properties relevant for T2D benefits.

**Figure 1:**
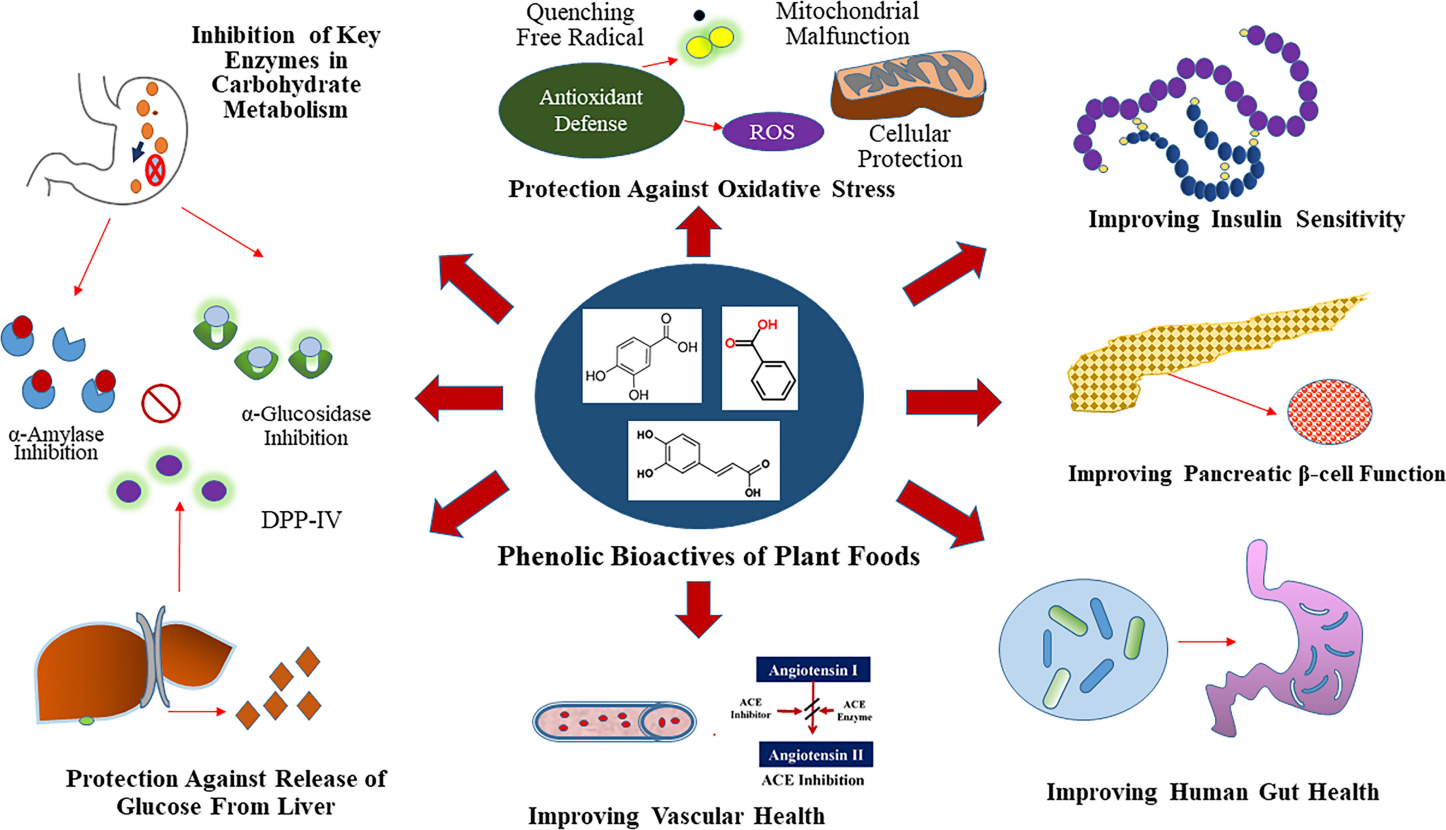
Glycemic Control Benefit Relevant Functional Properties of Plant Phenolics. Phenolics are dietary inhibitors of key enzymes involved in carbohydrate metabolism like α-amylase and α-glucosidase. Additionally, phenolics potentially inhibit dipeptidyl peptidase IV (DPP-IV), which is a dietary and therapeutic target to maintain glucose homeostasis. Select plant foods rich in phenolics also provide protection against chronic hypertension through inhibition of angiotensin-I-converting (ACE) enzyme.

Phenolic bioactives of plant foods have potential to modulate specific anti-hyperglycemic properties in small intestine, liver, muscle, adipose and pancreatic tissue (29). The anti-diabetic relevant anti-hyperglycemic functionalities of phenolics is due to their ability to modulate the activity of enzymes involved in glucose metabolism, as well as their ability to improve β cell function (insulin synthesis and secretion), reduce hepatic glucose output, and alter efficiency of glucose transporters and insulin receptors (8, 29) (**Figure 1**). Plant foods such as cocoa (*Theobroma cacao*), coffee (*Coffea* spp.), fruits, berries, nuts, vegetables, spices, medicinal herbs, tea (*Camellia sinensis*) and wine, that are a rich source of phenolic bioactives, have shown anti-hyperglycemic activity through different mechanisms of action, especially through the inhibition of the key enzymes like α-amylase, α-glucosidase, and aldose reductase (23, 33). Modification of the chemical structure of phenolic compounds *via* hydroxylation, methylation, glycosylation, hydrogenation or galloylation, can in turn increase or decrease the associated inhibitory activity against α-amylase and α-glucosidase enzymes (33). Modulation of activity of these key enzymes can help to slow down the breakdowns of complex carbohydrates and absorption of glucose into the bloodstream, which concurrently helps to maintain post-prandial glucose homeostasis leading to T2D benefits.

Additionally, phenolic metabolites can improve pancreatic β cell function (insulin synthesis and secretion) *via* activation of Sirtuin 1(Sirt 1) and glucose transporter 2 (Glut 2) through modulation of insulin dependent pathways that involves modifying cellular redox status and cell signaling activity (29, 32). Phenolics can also improve peripheral insulin sensitivity *via* activation of phosphoinositide 3-kinases (PI3K)/protein kinase B (AKT), Glut 4, and peroxisome proliferator-activated receptor gamma (PPAR-γ) in muscle, adipose and other tissues (29, 32). Furthermore, through insulin independent pathways, phenolics exhibit anti-hyperglycemic activity *via* activation of AMP-activated protein kinase (AMPK) in liver, muscle, and adipose tissues (29). The effect of phenolic compounds on these pathways are often correlated with improved β cell function and insulin sensitivity, reduced inflammation and lipotoxicity, as well as reduced hepatic glucose output and glucose absorption, all of which help to maintain normal glucose homeostasis (32). The molecular mechanisms by which phenolic compounds modulate activity against T2D can be broadly classified into four main categories that include transcriptional modulation, translational modulation, modulation of enzyme activity, and epigenetic regulation (34).

Another promising strategy to help lower postprandial blood glucose is through the inhibition of the enzyme dipeptidyl peptidase IV (DPP-IV), which inactivates the incretin hormones glucagon-like peptide-1 (GLP-1) and glucose-dependent insulinotrophic polypeptide (GIP), that are responsible for the stimulation of insulin secretion from pancreatic β cells as well as modulation of glucagon release from pancreatic α cells (35). Anthocyanins isolated from blueberry (*Vaccinium* spp.) and blackberry (*Rubus* spp.) wine blends, as well as several pure phenolic compounds like resveratrol, luteolin, apigenin, and flavone showed potent inhibitory activity against DPP-IV (36).

Therefore, phenolic metabolites that are widely distributed in plant foods have diverse anti-diabetic properties, which can be harnessed in functional food design and dietary strategies targeting wider T2D benefits. However, the phenolic profile and their anti-diabetic relevant functional properties vary widely among different plant foods, and plant parts as well as among different pre-harvest growing, post-harvest storage, food processing, and cooking conditions.

## Factors Influencing Phenolic Bioactive-Linked Antioxidant and Anti-Hyperglycemic Functionalities of Plant Foods

Consumers across the globe are increasingly making informed choices and integrating whole plant-based and minimally processed foods in their daily diet (37). However, the current food and nutrition model is mostly designed based on macro-nutrient content and whole calorie estimation and neglect the importance of other nutritional and bioactive components of plant foods like health protective phenolic bioactives (28). Therefore, to improve our understanding about nutritional and health protective functional benefits of foods, which will subsequently provide well-rounded nutritional information to consumers, it is necessary to know the phenolic bioactive composition and specific functional benefits of wide arrays of plant foods from different ecological origins. However, due to wide variability in the ecological diversity and distribution of phenolic bioactives in different plant food systems and even within different varieties/genotypes of same species, it can be challenging to design health-targeted foods with optimum phenolic level and composition (28). Additionally, as phenolics are stress inducible secondary metabolites of plants, pre and post-harvest factors like growing condition, cultivation practices, biotic and abiotic stress pressures, storage condition, different food processing and cooking methods significantly influence and alter the composition and overall content of phenolics in plant derived foods and beverages. Therefore, screening and optimization of different factors in the farm to fork food chain that have significant impact on phenolic bioactive linked functional quality of plant foods is essential to target them in health focused food solutions countering diet and lifestyle influenced T2D (**Table 1**).

**Table 1:** Antioxidant, anti-hyperglycemic, anti-hypertensive, and human gut health benefits of select plant foods from different geographic and ecological origins that are rich dietary sources for phenolic bioactives.

Plant Food Sources	Phenolic Content	Phenolic Profile	Antioxidant Property	Anti-Hyperglycemic Property	Anti-Hypertensive Property	Gut Health Benefits	Reference
***Cereals, Pseudo-Cereals, and Millets***							
Ancient Emmer Wheat with Hull (*In vitro*)	1.08-1.11 mg/g DW	*m*-Coumaric & gallic acids	0.65 mM Trolox Equivalent (TE)/g DW (ABTS)	88% α-amylase & 33-46% α-glucosidase inhibitory activities	n.d.	NA	(38)
Khorasan semolina diet	2.0 mg/g DW	NA	Increase in antioxidant capacity (6.3%)	9.1% reduction in blood glucose	NA	NA	(39)
Native Colored Corn from Peru (*In vitro*)	1.38-4.54 mg/g DW	*P*-coumaric, ferulic, caffeic acids & quercetin	18.8-67.7 µmol TE/g DW	13-29% α-amylase & 32-70% α- glucosidase inhibitory activities	n.d.	n.d.	(40)
Malting Barley (*In vitro*)	0.4-0.6 mg/g DW	Gallic, protocatechuic, caffeic acids & catechin	58-100% ABTS-based inhibition	60-97% α-amylase & 32-43% α- glucosidase inhibitory activities	n.d.	n.d.	(41)
Rye (*In vitro*)	1.2-2.9 mg/g DW	Ferulic, benzoic, protocatechuic, & gallic acids	95-97% ABTS-based inhibition & 43-54% DPPH based inhibition	71-89% α-amylase & 26-43% α- glucosidase inhibitory activities	NA	NA	(42)
Oat Groat (*In vitro*)	2.4 mg/g DW	Ferulic, benzoic, gallic acids & catechin	80-88% ABTS-based inhibition & 15-28% DPPH based	40-47% α-amylase & 17-38% α- glucosidase inhibitory activities	n.d.	*Helicobacter pylori* inhibition	(43)
Buckwheat (*In vitro*)	2.8 mg/g DW	*P*-coumaric, gallic, ferulic, protocatechuic acids, catechin, & quercetin	80% ABTS-based inhibition & 67% DPPH based	48% α-amylase & 26% α- glucosidase inhibitory activities	n.d.	n.d.	
Teff (*In vitro*)	1.8 mg/g DW	*P*-coumaric, gallic, protocatechuic acids, catechin, & rutin	92% ABTS-based inhibition & 58% DPPH based inhibition	25% α-amylase &30% α- glucosidase inhibitory activities	n.d.	n.d.	
Sorghum (*In vitro*)	1.1-9.7 mg/g DW	*P*-coumaric, gallic, benzoic, protocatechuic, cinnamic, benzoic acids, & catechin	48-97% ABTS-based inhibition 17-85% DPPH based inhibition	6-48% α-amylase & 6-99% α- glucosidase inhibitory activities	n.d.	n.d.	
Pearl Millet (*In vitro*)	1.9 mg/g DW	*P*-coumaric, gallic, benzoic, protocatechuic & cinnamic acids	75% ABTS-based inhibition 46% DPPH based inhibition	20% α-amylase & 10% α- glucosidase inhibitory activities	NA	NA	
Red Quinoa	3.0 mg/g DW	Quercetin	88% DPPH based inhibition	32% α- glucosidase inhibitory activity	NA	NA	(44)
Kaniwa	2.3 mg/g DW	Quercetin	72% DPPH based inhibition	18% α- glucosidase inhibitory activity	NA	NA	
***Beans and Legumes***							
Black bean	3.0-4.5 mg/g DW	NA	22-81% DPPH based inhibition	80-85% α-amylase (half diluted) & 22-35% α- glucosidase inhibitory activities	NA	NA	(45)
Andean Legume (*Lupinus mutabilis*)	4.0 mg/g DW	n.d.	40% DPPH based inhibition	20-30% α- glucosidase inhibitory activity	55% ACE inhibition	NA	(44)
Snap Bean	0.2-0.3 mg/g FW	NA	40-80% ABTS based inhibition	25-90% α-amylase & 26-52% α- glucosidase inhibitory activities	NA	NA	(46)
Peruvian and Brazillian Dry Bean	2.5-6 mg/g FW	Chlorogenic, & caffeic acids	30-85% DPPH based inhibition	90% α-amylase & 15-35% α- glucosidase inhibitory activities	80% ACE inhibition		(47)
***Vegetables and Root Crops***							
Pepper	0.7-2 mg/g FW	NA	10-80% DPPH based inhibition	0-75% α-amylase & 25-58% α- glucosidase inhibitory activities	20-80% ACE inhibition	NA	(48)
Eggplant	20-75 µg/mL	NA	5-45% DPPH based inhibition	20-30% α-amylase & 40-55% α- glucosidase inhibitory activities	0-70% ACE inhibition	NA	(49)
Potato (Chilean varieties)	1-12 mg/g DW	Chlorogenic, ferulic, caffeic, p-coumaric acids, & catechin	10-80% DPPH based inhibition	10-50% α- glucosidase inhibitory activity	0-85% ACE inhibition	NA	(50)
Kale	2.3 mg/g FW	Ferulic, dihydroxy-benzoic, & cinnamic acids	90% ABTS based inhibition	48% α- glucosidase inhibitory activity	85% ACE inhibition	NA	(51)
Beetroot	0.7 mg/g FW	Gallic & benzoic acids	88% ABTS based inhibition	15% α- glucosidase inhibitory activity	72% ACE inhibition	NA	
Carrot	0.3 mg/g FW	Dihydroxybenzoic acid	80% ABTS based inhibition	36% α- glucosidase inhibitory activity	30% ACE inhibition	NA	
Sweet Potato	0.5-2.7 mg/100g FW	Ferulic, dihydroxy-benzoic, chlorogenic, gallic acids & catechin	0.5-0.6 mM Trolox Equivalent/g FW ABTS based inhibition	60-90% α-amylase & 20-60% α- glucosidase inhibitory activities	10-80% ACE inhibition	NA	(52)
***Fruits***							
Apple varieties (peel & pulp)	0.2-0.8 mg/g FW	Chlorogenic, protocatechuic, *p*-coumaric acids, & quercetin	20-80% DPPH based inhibition	20-100% α-amylase & 30-85% α- glucosidase inhibitory activities	NA	NA	(53)
Pear (Bartlett & Starkrimson peel and pulp)	0.2-1.3 mg/g FW	Chlorogenic, protocatechuic, gallic, caffeic, *p*-coumaric acids, & catechin	20-70% DPPH based inhibition	80-99% α-amylase & 60-90% α- glucosidase inhibitory activities	25% ACE inhibition (Bartlett)	*Helicobacter pylori* inhibition	(54)
Cherry	0.6 mg/mL	Caffeic, *p*-coumaric acids, & epicatechin	80% DPPH based inhibition	50-80% α- glucosidase inhibitory activity	0-40% ACE inhibition (Bartlett)	*Helicobacter pylori* inhibition	(55)
Camu-Camu	0.2-1.2 mg/mL	Ellagic acid, ellagitannins, quercetin, & myrecetin	20-60% DPPH based inhibition	20-85% α-amylase & 90-100% α- glucosidase inhibitory activities	75-80% ACE inhibition	NA	(56, 57)
Jambolan	5.5 mg/g DW	NA	60% DPPH based inhibition	44-85% α-amylase & 63-99% α- glucosidase inhibitory activities	NA	NA	(58)
Persimon	4.6 mg/g DW	NA	50% DPPH based inhibition	29-88% α-amylase & 75-86% α- glucosidase inhibitory activities	NA	NA	
Plum	6.4 mg/g DW	NA	70% DPPH based inhibition	3-90% α-amylase & 20-93% α- glucosidase inhibitory activities	NA	NA	
Red Grape	7.0 mg/g DW	NA	75% DPPH based inhibition	88% α-amylase & 44-98% α- glucosidase inhibitory activities	NA	NA	
Black Grape	3.0 mg/g DW	NA	30% DPPH based inhibition	87% α-amylase & 45-54% α- glucosidase inhibitory activities	NA	NA	
Jabuticaba	7.5 mg/g DW	NA	90% DPPH based inhibition	30-93% α-amylase & 78-94% α- glucosidase inhibitory activities	NA	NA	
Surinam Cherry	7.0 mg/g DW	NA	85% DPPH based inhibition	0-74% α-amylase & 99-100% α- glucosidase inhibitory activities	NA	NA	
***Berries***							
Blueberry	1-1.4 mg/g FW	Catechin, gallic, protocatechuic acids, & quercetin	60-80% DPPH based inhibition	50-90% α-amylase & 60-80% α-glucosidase inhibitory activities	NA	NA	(59)
Blackberry	1.2-2.0 mg/g FW	Gallic, benzoic, *p*-coumaric, caffeic acids, rutin, & catechin	60-90% DPPH based inhibition	40-90% α-amylase & 70-99% α- glucosidase inhibitory activities	NA	NA	(60)
Red & Black Currant	2.5-13 mg/g FW	Chlorogenic, p-coumaric, protocatechuic acids, & quercetin	50-65% DPPH based inhibition	10-85% α-amylase & 65-99% α- glucosidase inhibitory activities	10-30% ACE inhibition	NA	(61)
***Medicinal Plants, Herbs, and Spices***							
Mint	36 mg/g DW	Rosmarinic, ellagic, benzoic, & gallic acids	59% DPPH based inhibition	84% α- glucosidase inhibitory activity	n.d.	NA	(62)
Basil	1.0-6.0 mg/g FW		27-84% DPPH based inhibition	10% α-amylase & 50-90% α- glucosidase inhibitory activities	n.d.	n.d.	
Dill	6.5 mg/g DW	NA	75% DPPH based inhibition	25% α-amylase & 50% α- glucosidase inhibitory activity	n.d.	n.d.	(63)
Fennel	5.8 mg/g DW	NA	72% DPPH based inhibition	15% α-amylase & 15% α- glucosidase inhibitory activities	n.d.	n.d.	
Caraway	4.6 mg/g DW	NA	65% DPPH based inhibition	30% α-amylase & 18% α- glucosidase inhibitory activities	n.d.	n.d.	
Coriander	4.2 mg/g DW	NA	70% DPPH based inhibition	15% α-amylase & 0-10% α- glucosidase inhibitory activities	n.d.	n.d.	
Anise	5.5 mg/g DW	NA	58% DPPH based inhibition	20% α-amylase & 0-10% α- glucosidase inhibitory activities	n.d.	n.d.	
Ajowan	8.2 mg/g DW	NA	85% DPPH based inhibition	50% α- glucosidase inhibitory activity	n.d.	n.d.	
*Rhodiola*	124 mg/g DW	Gallic, *p*-coumaric acid, & Tyrosol	75% DPPH based inhibition	10% α-amylase & 0-90% α- glucosidase inhibitory activities	10% ACE inhibition	NA	(64)
*Swertiya chirayita*	15 mg/g DW	Mangiferi, swertiamerin, & amarogentin	70-80% DPPH based inhibition	20-40% α- glucosidase inhibitory activity	n.d.	n.d.	(65)

n.d, not detected.

NA, Not Applicable (Not targeted in the study).

ACE- angiotensin-I-converting enzyme.

### Screening of Food Plant Species and Varieties/Genotypes

#### Cereals, Pseudo-Cereals, and Millets

Cereals, pseudo-cereals, and millets are major sources of carbohydrates in the human diet, and they support the essential calorie needs as well as play a critical role in addressing food and nutritional insecurity solutions, especially to counter chronic hunger and malnutrition globally. However, higher intake of hyper-processed and refined cereals as simple sources of dense soluble macronutrient carbohydrates also increases the risks of developing T2D (66). Therefore, there is a growing interest to integrate whole grain cereals with balanced nutritional profile and higher concentration of fiber and health protective bioactives for optimum dietary benefits and health protection. The current scientific evidence clearly indicates that there is a substantial inverse relationship between higher consumption of whole grains and risks of developing T2D (67). The anti-hyperglycemic benefits of whole grains are associated with low energy intake, prevention of weight gain, and has direct influence on insulin resistance (68). The higher dietary fiber content and phenolic bioactive content of whole grain is also relevant in supporting beneficial gut microbiome as well as providing protection against chronic oxidative stress, inflammation, and hyperglycemia (69).

*In vitro* studies have shown that ancient accessions of cereal grains, like ancient wheat (emmer- *Triticum dicoccon*, einkorn-*Triticum monococcum* and spelt-*Triticum spelta*) have wider diet-linked health benefits such as capacity to reduce ROS formation, lower blood glucose and cholesterol levels, and reduce circulating vascular endothelial growth factors and interleukins levels, which are relevant for countering T2D (39, 70). In a recent *in vitro* model based screening study, higher phenolic linked antioxidant and anti-hyperglycemic properties (high α-amylase and moderate α-glucosidase enzyme inhibitory activities) were observed in emmer wheat (*Triticum dicoccon*) with hull both before and after milling, when compared to modern wheat (*Triticum aestivum*) varieties (Barlow and Coteau) (38). The emmer wheat with hull had significantly high *m*-coumaric acid, which was not present in dehulled emmer and modern wheat varieties (38). Like the results of ancient wheat, higher phenolic content and associated antioxidant and anti-hyperglycemic functional qualities were observed in different colored corn (*Zea mays*) races and accessions, which are indigenous native corn of North and South America (40, 71). Colored corn accessions with high anthocyanin content and high antioxidant activity also exhibited high α-glucosidase and lipase enzyme inhibitory activities (40). In Peruvian purple corn accession (*Zea mays* subsp*. indurata*), higher concentration of anthocyanin, hydroxy-cinnamic acids like *p*-coumaric acid and caffeic acid derivatives were found as free phenolic fractions, while ferulic acid was the predominant bound phenolic fraction in these colored corn accessions (71).

High antioxidant and anti-hyperglycemic properties were also observed in other cereal grains with high phenolic bioactive content like barley (*Hordeum vulgare*), rye (*Secale cereale*), and oat (*Avena sativa*) (41–43, 72). Among different barley (*Hordeum vulgare*) varieties, high anti-hyperglycemic property relevant α-glucosidase and lipase enzyme inhibitory was found in black barley type, while high phenolic-linked antioxidant activity was observed in malting barley cultivar Pinnacle (41). Overall, barley is a good dietary source of phenolic compounds and other functional constituents like β-glucans (insoluble and soluble fiber) and can potentially play health protective role in mitigating T2D associated risk factors (73). Like barley, rye (*Secale cereale*) is also rich source of phenolic bioactives and dietary fiber and has exhibited anti-cholesterol and glycemic control relevant properties (42). In a previous *in vitro* screening study, high total soluble phenolic content, high antioxidant activity, very high α-amylase and moderate to high α-glucosidase enzyme inhibitory activities were observed in rye varieties (42). High ferulic acid, benzoic acid, protocatechuic acid, and gallic acid content were found in the aqueous food grade extracts of most rye varieties (42). In another study, high phenolic-linked antioxidant and α-amylase enzyme inhibitory activity was observed in organic whole grain oat (*Avena sativa*) (43). Additionally, same whole grain oat extracts also exhibited inhibitory activity against ulcer causing bacteria *Helicobacter pylori* (43). Therefore, barley, rye, and whole grain oat is addition to being good dietary sources of phenolic antioxidants are relevant for managing chronic oxidative stress and chronic hyperglycemia and also have potential to counter human gut infection, which is pertinent for addressing T2D associated gut infection.

In addition to whole grain oat, higher antioxidant and anti-hyperglycemic properties were observed in buckwheat (*Fagopyrum esculentum*), teff (*Eragrostis tef*), and pearl millet (*Pennisetum glaucum*) (43). The results of phenolic compound characterization revealed that ferulic acid and catechin were major phenolic compounds in whole grain oat and buckwheat, while teff and pearl millet had higher protocatechuic acid and gallic acid (43). Similarly, very high phenolic content and positive correlation with antioxidant and α-glucosidase enzyme inhibitory activity was observed in different sorghum (*Sorghum bicolor*) genotypes (43). Cinnamic acid was the predominant phenolic bioactive detected in all sorghum genotypes (43). Previously, higher content of quercetin and very high antioxidant activity were found in pseudo-cereals, Quinoa (*Chenopodium quinoa*) and Kaniwa (*Chenopodium pallidicaule*) of Peruvian Andean origin (44). Results of these previous screening studies indicated that whole grain cereals, especially ancient cereal grains with cultural and ecological relevance are rich sources of health protective phenolic bioactives and can be rationally integrated in dietary and therapeutic interventions to counter chronic inflammation, hyperglycemia, and gut infection associated with T2D.

#### Beans and Other Legumes

The current scientific evidence clearly suggests that legumes with balanced profiles of dietary fiber, micronutrients, and phenolic bioactives in a high protein matrix have human metabolism-linked protective function such as favorable glucose metabolism related benefits. Previously, high phenolic bioactive associated antioxidant and anti-hyperglycemic properties were found in black bean (*Phaseolus vulgaris*) varieties (45). Black beans are also a rich source of micronutrients including zinc, essential amino acids, and dietary fibers and has potential to support healthy immunity and wider functional benefits that are relevant to counter malnutrition, infection as well as different metabolic disorders (74).

In a recent screening study, high phenolic-linked antioxidant, α-amylase, and α-glucosidase enzyme inhibitory activities were observed in different snap bean (*Phaseolus vulgaris*) genotypes (46). Higher content of quercetin, catechin, and gallic acid were also found in all snap bean genotypes (46). Similarly, Randhir et al. (75) observed high antioxidant, anti-hyperglycemic, and anti-hypertensive property relevant functional qualities in thermally processed select legume sprouts and seedlings (fava bean-*Vicia faba*, mung bean-*Vigna radiata*, and soybean-*Glycine max*). In another study, high antioxidant, and moderate anti-diabetic (α-amylase, and α-glucosidase enzyme inhibition) and low anti-hypertensive (ACE inhibition) property were found in Peruvian and Brazilian bean (*Phaseolus vulgaris*) varieties (47). All these studies suggest that traditional as well as modern legume varieties with rich phenolic bioactive content in high protein matrix are excellent dietary targets to address chronic oxidative stress, hyperglycemia, and hypertension, which are major risk factors of T2D.

#### Vegetables and Root Crops

Like legumes, vegetables are also extremely relevant dietary targets to address current diet and lifestyle influenced T2D challenges. Higher intake of vegetables has been associated with a reduced incidence of T2D and can help to reduce other NCD related health risks (76, 77). In addition to phenolic bioactives, vegetables are also rich source of dietary fiber, folate, and several micronutrients, which are relevant for diverse human health protective functional qualities including oxidative stress protection, glycemic control, and supporting human digestive health. A dose response meta-analysis-based cohort study indicated that 2-3 servings/day of vegetables were associated with lower risk of T2D (78). In another study, reduction of T2D by 9% was observed with increase in intake of vegetables up to 300 g/day (79).

One of the major plant families with many popular vegetable crops is *Solanaceae*, which include pepper (*Capsicum* spp.), eggplant (*Solanum melongena*), tomato (*Solanum lycopersicum*), and potato (*Solanum tuberosum*). Previously, Kwon et al. (48) screened several types of pepper (*Capsicum annuum*) (green, red, orange, yellow, Cubanelle, red sweet, yellow sweet, long hot and Jalapeño) and found high α-glucosidase and low α-amylase enzyme inhibitory activities in aqueous extracts. In the same study, ACE inhibitory activity was also observed in select peppers. Potential anti-diabetic benefits of eggplant (*Solanum melongena*) were also examined using *in vitro* assay models (49). Eggplant varieties with high phenolic content exhibited very high α-glucosidase and moderate to high ACE enzyme inhibitory activities. Like eggplant, extracts of Chilean potato (*Solanum crispum*) of diverse colors also showed moderate α-glucosidase and moderate to high ACE inhibitory activity, relevant for managing chronic hyperglycemia and hypertension (50).

In a recent study, similar antioxidant, antidiabetic, and anti-hypertensive benefit relevant functional qualities were observed in vegetables, which were targeted as part of Native American “Three Sisters Crop” community garden concept (51). Very high phenolic bioactive-linked antioxidant, moderate α-glucosidase, and high ACE enzyme inhibitory activity was found in kale (*Brassica oleracea* var. *sabellica*) followed by purple potato (*Solanum tuberosum*), beetroot (*Beta vulgaris*), pepper (*Capsicum annuum*), and carrot (*Daucus carota*) (51). Higher concentration of dihydroxybenzoic acid, ferulic acid, and cinnamic acid were found in kale extracts, which are potentially relevant for its high antioxidant, anti-hyperglycemic and anti-hypertensive functional qualities (51). A recent screening study with different colored (white, orange, and purple) sweet potato (*Ipomoea batatas*) varieties found high phenolic bioactive linked antioxidant activity in purple fleshed variety, while high anti-hyperglycemic (α-amylase and α-glucosidase enzyme inhibition) and anti-hypertensive properties (ACE inhibition) were found in orange and white-fleshed sweet potato varieties (52). All these screening studies indicated that vegetables with rich source of phenolic bioactives are good dietary targets to counter chronic hyperglycemia and oxidative stress induced metabolic breakdowns as well as potentially help to manage chronic hypertension, which is a common health complication of T2D.

#### Fruits

Fruits are rich sources of stress protective bioactive compounds and other essential nutrients with diverse health benefits. Therefore, higher consumption of fruits with rich dietary antioxidant profiles is also relevant in countering oxidative stress-induced metabolic breakdowns and related chronic diseases like T2D. In a prospective longitudinal cohort study, Muraki et al. (80) found that higher intake of specific whole fruits, like blueberry (*Vaccinium* spp.), grapes (*Vitis vinifera*), and apple (*Malus domestica*) was positively correlated with reduced risk of T2D, and high dietary fiber and bioactive antioxidant content of these fruits might have contributed towards such anti-diabetic relevant benefits. In a clinical study, reduction of HbA_1C_, low blood pressure and reduced risk of coronary heart disease were observed with higher consumption of fruits with low glycemic index such as apple (*Malus domestica*), orange (*Citrus × aurantium*), pear (*Pyrus communis*), tangerine (*Citrus reticulata*), blueberry (*Vaccinium* spp.), strawberry *(Fragaria × ananassa*), nectarine (*Prunus persica* var. *nucipersica*), plum (*Prunus domestica*), peach (*Prunus persica*), grapefruit (*Citrus paradisi*), raspberry (*Rubus idaeus*), blackberry (*Rubus* spp.), and breadfruit (*Artocarpus altilis*) (81). In a recent meta-analysis of prospective cohort studies revealed that increase in consumption at a rate of one serving of apple and pear per week resulted into 3% reduction of T2D risk (82). Both apple and pear are good sources of dietary fiber and natural antioxidants like flavonoids and phenolic acids and have shown anti-hyperglycemic and anti-hypertensive properties relevant for dietary interventions to counter T2D associated complications (83).

Significant impact of varietal influence on phenolic-linked antioxidant and *in vitro* anti-hyperglycemic properties were observed in select apple varieties (53). Additionally, higher phenolic content, antioxidant activity, and α-glucosidase enzyme inhibitory activity were found in well preserved apple (Red Delicious, Honey Crisp, Cortland) peel extracts, while pulp extracts exhibited higher α-amylase enzyme inhibitory activity (53, 84). The major phenolic compounds found in apples are protocatechuic acid, chlorogenic acid, *p*-coumaric acid, and gallic acid. Similarly, carbohydrate enzyme modulating properties (α-glucosidase and α-amylase enzyme inhibitory activities) relevant to counter chronic hyperglycemia were observed in varieties (Mcintosh, Empire, Cortland, and Mutsu) of well-preserved stored apple (85).

High phenolic-linked antioxidant and anti-hyperglycemic functional properties were also observed in pear (54). Additionally, anti-hypertensive property relevant ACE inhibitory activity was observed in pulp extracts of Bartlett pear (54). Another previous *in vitro* study (86), also reported similar phenolic-linked antioxidant, anti-hyperglycemic (α-glucosidase and α-amylase enzyme inhibitory activities), and anti-hypertensive (ACE inhibition) properties in freshly harvested and long term stored pear varieties (Bartlett, Bosc, Comice, Seckel, Red D’ Anjou, and Green D’ Anjou). Major phenolic compounds found in pear peel were *p*-coumaric acid, protocatechuic acid, caffeic acid, and gallic acid, while pear pulp was a good source of chlorogenic acid, catechin and gallic acid (54). Another Rosaceae family fruit, cherry (*Prunus cerasus*) is also rich source of bioactive phenolics including anthocyanin. Inhibition of α-amylase, α-glucosidase, cyclooxygenase-1, lipoxygenase, cyclooxygenase-2, and xanthine oxidase were observed with tart cherry (Montmorency) extracts (87). Above studies indicated that whole fresh and well-preserved fruits like apple, pear, and cherry are a rich source of phenolic antioxidants that have diverse health protective functional qualities such as improving glucose metabolism and overall glycemic control.

In addition to these popular fruits, underutilized and more localized fruits from diverse ecological and geographical origin across the globe have also shown anti-diabetic and cardiovascular health protective properties. In a study with native Peruvian fruits, Pinto et al. (88) found high α-glucosidase enzyme inhibitory activity and a positive correlation with phenolic content and antioxidant activity in food grade aqueous extracts of Lucuma (*Pouteria lucuma*) and Algarrobo (*Prosopis pallida*). Additionally, high ACE inhibitory activity was observed in Papayita arequipeňa (*Vasconcellea pubescens*) and Algarrobo relevant for managing chronic hypertension associated with type 2 diabetes and cardiovascular diseases (88). Very high *in vitro* α-glucosidase enzyme inhibitory activity and high antioxidant potential were observed in another Amazonian fruit, camu-camu (*Myrciaria dubia*) (56). In an *in vitro* screening study, high anti-hyperglycemic (α-amylase and α-glucosidase enzyme inhibitory activities) and high antioxidant properties and their proportional positive correlation with soluble phenolic content were observed in peel, pulp, and seed extracts of select Brazilian fruits (Jabuticaba- *Plinia peruviana*, Surinam Cherry- *Eugenia uniflora*, Jambolan plum-*Syzygium cumini*, Persimmon-*Diospyros kaki*, plum- *Prunus domestica*, red grape- *Vitis vinifera* L., and black grape-*Vitis labrusca*) (58). All these screening studies indicate that different parts of fruits (peel, seed, and pulp) are rich sources of a wide array of phenolic bioactives with high antioxidant and glucose metabolism modulating potential and can be targeted in dietary and therapeutic support strategies to improve glycemic control, and subsequently reduce the risk of T2D.

#### Berries

The superior dietary and nutritional benefits of berries are mostly attributed to their unique health promoting phenolic bioactive profile and related high antioxidant activity. Berries are a good source of dietary antioxidants like anthocyanin, flavonoids, and ellagitannins and can be integrated into fresh, frozen, and processed foods in dietary and therapeutic support strategies to counter chronic oxidative stress associated diseases. Beyond their high antioxidant property, berries also have wider human health protective functional qualities including potential for improving glucose metabolism and glycemic control and for providing protection against lipid and protein oxidation (60, 89, 90).

Consistent body of previously published literature based on both *in vitro* and *in vivo* studies have revealed high phenolic bioactive-linked antioxidant and anti-hyperglycemic (α-amylase and α-glucosidase enzyme inhibitory activities) functional qualities in berries (59–61, 90–95). Among different berries, strawberry is one of the most popular choices among consumers and demand for this fruit as a fresh and processed food is increasing rapidly. High α-glucosidase, moderate α-amylase, and moderate ACE enzyme inhibitory activities were observed in aqueous and ethanolic extracts of select strawberry *(Fragaria × ananassa*) varieties (92). In another *in vitro* study, anti-proliferative, anti-hyperglycemic, and anti-hypertensive properties of purified ellagitannins from strawberries were investigated and compared with ellagic acid and strawberry extracts (94). High anti-proliferative activity was observed with ellagic acid, while purified ellagitannins from strawberry had high α-amylase and ACE inhibitory activities (94).

Like strawberry, glucose metabolism supporting functional benefits relevant for anti-diabetic application were also observed in blueberries (59, 93, 95, 96). In an *in vitro* model-based screening study to understand the effect of highbush blueberry fruit developmental stages on phenolic-linked anti-hyperglycemic functional qualities, higher soluble phenolic content and high chlorogenic acid content were found in green fruit (before completely ripe), while extracts of fully ripe berries had high antioxidant, high α-glucosidase and moderate α-amylase enzyme inhibitory activities (93). Similarly, significant impact of different varieties/genotypes on phenolic bioactive-linked (based on positive correlation between parameters) antioxidant and glucose metabolism relevant functional benefits (high *in vitro* α-amylase and α-glucosidase enzyme inhibitory activities) were also observed in blueberries (*Vaccinium* spp.) (59). The major phenolic compounds found in food grade aqueous extracts of these varieties were catechin, quercetin, protocatechuic acid, and gallic acid (59). High phenolic bioactive-linked antioxidant and anti-hyperglycemic functional properties as well as significant impacts of different varieties/genotypes on these health-targeted qualities were also found in blackberry (*Rubus* spp.) (60, 97).

Similarly, significant *in vitro* α-glucosidase and ACE enzyme inhibitory activities were observed in cranberry (*Vaccinium microcarpum*) powders having high quercetin and *p*-coumaric acid content (98). In another *in vitro* screening study, da Silva Pinto et al. (61) found moderate to high α-glucosidase, α-amylase, and ACE enzyme inhibitory activities in red (*Ribes rubrum*) and black currant (*Ribes nigrum*) varieties. Recently, Espe (97) observed very high phenolic content, antioxidant, anti-hyperglycemic, and anti-hypertensive properties in several genotypes of serviceberry/juneberry (*Amelanchier* spp.) grown under cold temperate climate in the Northern Great Plains of the United States. Phenolic enriched berries have also shown other health protective functional qualities such as protection against gut microbiome dysbiosis and dyslipidemia, the common risk factors associated with T2D (99, 100). Due to such high source of dietary antioxidants and associated glucose metabolism supporting functional properties, metabolically screened and optimized berries can be incorporated into food synergies and functional food design for anti-diabetic benefits.

#### Medicinal Herbs and Spices

Medicinal herbs and spices have long been used in traditional medicine for wider health benefits, including managing post-prandial blood glucose levels, which is essential to counter chronic hyperglycemia and associated metabolic breakdowns of T2D. Among popular and common medicinal herbs, plants from the family *Lamiaceae* have been found in every region of the world and are widely used in food and therapeutic applications for centuries (62). Medicinal herbs from this plant family are high in biphenolics, like rosmarinic acid and have a wide range of flavonoids and mono-phenolics (62). In a recent *in vitro* study, high phenolic bioactive linked antioxidant and α-glucosidase enzyme inhibitory activity were found in food grade extracts of select clonal lines of Asian basil (*Ocimum* spp.) (62).

Another important plant family with health relevant therapeutic properties is *Apiaceae*, which has many common spices and condiments like coriander (*Coriandrum sativum*), caraway (*Carum carvi*), anise *(Pimpenella anisum*), fennel (*Foeniculum vulgare*), dill (*Anethum graveolens*), and ajowan (*Trachyspermum ammi*). Moderate to high α-glucosidase, moderate α-amylase, and high antioxidant activity were observed in seed extracts of dill, fennel, caraway, coriander, and anise, while ajowan extracts showed high antioxidant and moderate α-glucosidase enzyme inhibitory activity (63). Similarly, crude ethanol extracts of cumin (*Cuminum cyminum*) seeds with high phenolic and flavonoid content exhibited reduction in glycemic level and inflammation, as well as improved plasma lipid profile in diabetes induced rats (101).

There are many medicinal plants, herbs, and spices from diverse geographical and ecological origins, especially from rich biodiversity regions (Himalayas, Andes, Amazon, Western Ghats of India and Borneo in South East Asia), that have shown wider anti-diabetic and human health protective functional properties. High *in vitro* α-glucosidase and moderate ACE enzyme inhibitory activities were observed in aqueous and ethanolic extracts of *Rhodiola crenulata* and *Rhodiola rosea*, a rich source of tyrosol and other phenolic bioactives (102). Similarly, moderate inhibition of α-glucosidase enzyme and its positive correlation with phenolic content was also observed in ethanolic extracts of *Ginkgo biloba* leaf (103). Medicinal herbs and spices from Andean region like Chancapiedra (*Phyllanthus niruri* L.), Zarazaparrilla (*Smilax officinalis*), Yerba Mate (*Ilex paraguariensis* St-Hill), and Huacatay (*Tagetes minuta*) have shown high antioxidant and high α-glucosidase enzyme inhibitory activity in an *in vitro* model based screening study (104). In another study, flower, and leaf extracts of Loquat (*Eriobotrya japonic*a Lindl.) with high chlorogenic acid and quercetin content exhibited significantly high anti-hyperglycemic activity (α-amylase and α-glucosidase enzyme inhibitory activities) (105). Crude extracts of a medicinal plant *Swertia chirayita* from Himalayas (Nepal) showed high phenolic-linked antioxidant and moderate α-glucosidase enzyme inhibitory activities (65). Mangiferin, swertiamarin, and amarogentin were major phenolic compounds found in extracts of *Swertia chirayita* (65). All these screening studies indicate that many medicinal plants, herbs, and spices are rich sources of phenolic bioactives with wider glycemic control benefits (**Table 1**).

### Impact of Cultivation Practices and Growing Environments

**Figure 2:**
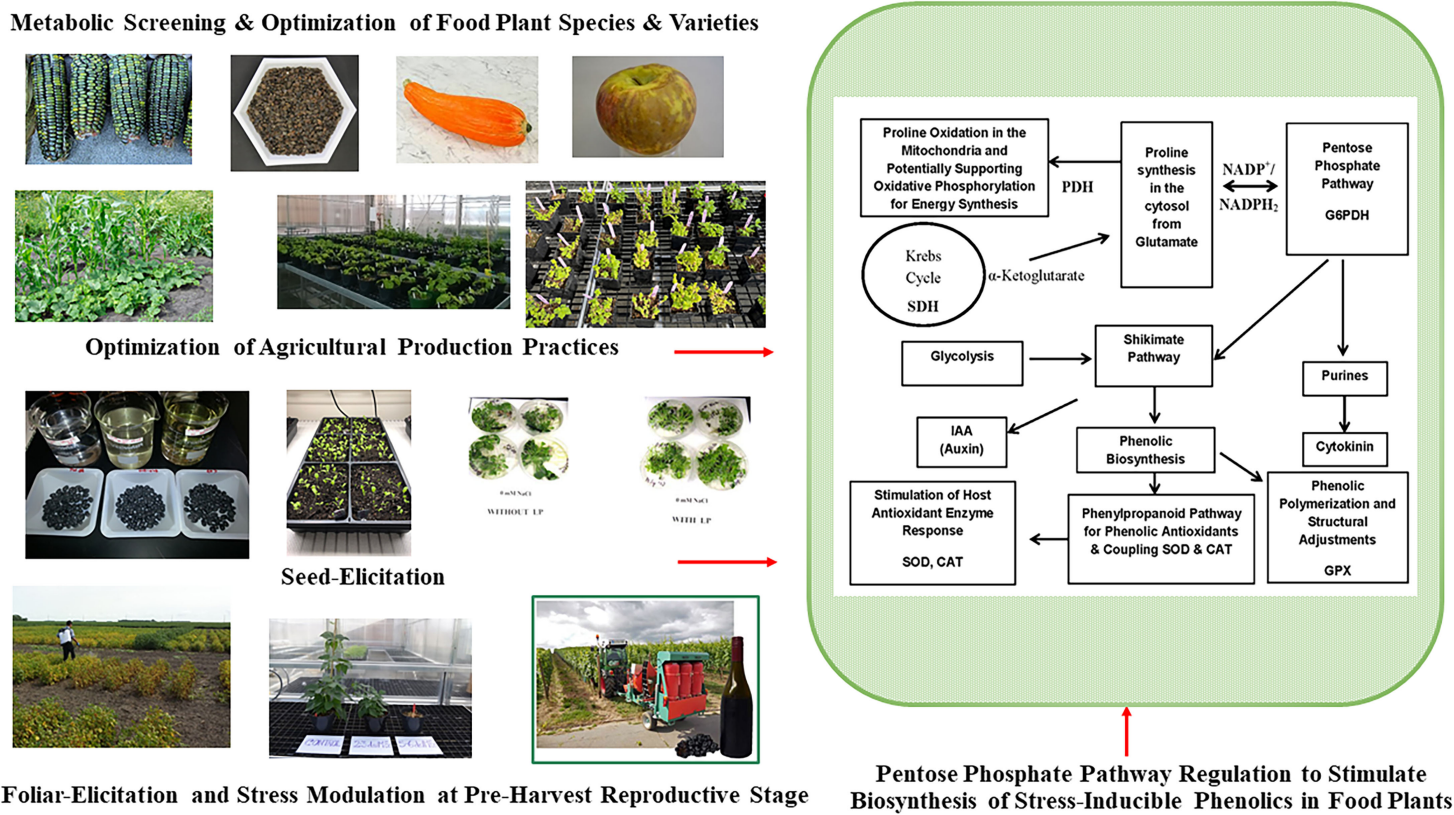
Pre-Harvest Stress Inducible Strategies to Stimulate Biosynthesis of Human Health Protective Phenolic Bioactives in Food Plants. Mild stress induction at critical reproductive stage drive carbon flux towards biosynthesis of stress protective metabolites like phenolics through upregulation of pentose phosphate pathway (PPP), shikimate pathway, and phenylpropanoid pathway. Metabolically driven screening also help to find plant foods with optimum phenolic bioactive profile and associated anti-diabetic functionalities. Glucose-6-phopshate dehydrogenase (G6PDH); proline dehydrogenase (PDH); succinate dehydrogenase (SDH); superoxide dismutase (SOD), catalase (CAT), guaiacol peroxidase (GPX).

Phenolic metabolites of food and medicinal plants are stress-inducible, and their composition, content, and health protective functional qualities vary widely due to differences in growing environment and across different agricultural and horticultural production practices (**Figure 2** and **Table 2A**). Variations of such stress inducible phenolic metabolites impose significant challenges to consistently find plant-based edible materials with uniform consistency and optimum bioactive profile and associated functional qualities relevant for health-focused dietary solutions (28). There is limited understanding and insufficient empirical evidence to a find clear relationship between agricultural production practices and specific health targeted phenolic bioactive-linked nutritional qualities of plant foods. One of the rapidly growing agriculture markets is organic food, which is mostly driven by a wider perception among consumers about their superior nutritional and sensory qualities. However, many previous studies were inconclusive and did not find significant differences in bioactive phenolic content and associated nutritional qualities of plant foods between organic and conventional production systems (143–145).

**Table 2A:** Pre-harvest strategies to improve the glycemic control-relevant bioactivity of plant-based foods and beverages.

Pre-Harvest Stress-Modulating Treatments	Physical, Chemical or Biological Agent	Concentration, Temperature or Time	Plant-based Food or Beverage	Application	Improved Human Health-Relevant Activities	Reference
Physical treatment	Heat shock	140°C	Red wine	Foliar application after bud formation	Antioxidant & Anti-hyperglycemic activity	(106)
Chemical Treatment	Sodium chloride	0 to 32 dS m−1	Common Purslane	Addition of saline water 30 days after transplantation of seedlings	Phenolic content, & antioxidant activity	(107)
	Ozone	25 gal per acre	White & red grapes	Foliar application every 10 days	Phenolic content and Anti-hyperglycemic activity	(108)
	Salicylic acid & acetyl salicylic acid.	0.5 to 2.0 mM	Cherries	Foliar application at 98, 112, and 126 days after full bloom	Phenolic content and antioxidant activity	(109)
Biological treatment	Chitosan oligosaccharide (COS)	5 g/L	Black beans	Foliar application during flowering stage	Phenolic content, antioxidant & anti-hyperglycemic activity	(45)
		0.05 to 1 g/L.	Greek oregano	Foliar application before flowering stage	Phenolic content	(110)
		50 mg/L	Strawberry	Foliar application at seedling, before flowering, fruit coloring, and full bloom stage	Phenolic content and antioxidant activity	(111)
	*Aspergillus niger* & *Rhizophus oryzae*.	5 to 10% conc.	Dates (*Phoenix dactylifera*)	Foliar application after pollination and fruit setting stage	Antioxidant activity	(112)

**Table 2B:** Post-harvest strategies to improve the glycemic control-relevant bioactivity of plant-based foods and beverages.

Post-Harvest Treatments	Physical, Chemical or Biological Agent	Concentration, Temperature or Time	Plant-based Food or Beverage	Stress Treatment, Processing and Fermentation	Improved Human Health-Relevant Activities	Reference
Physical treatment	Temperature & irradiance	21 to 32°C 5 or 200 μmol m^-2^ s^-1^.	Tomato	Exposure of fruit at day 1 to 8 of ripening	Phenolic content	(113)
	Thermal processing	121°C for 20 min	Buckwheat, wheat, & oats (sprouts or seedlings)	Autoclaving of sprouts (day2) and leaves (day 10)	Phenolic content, Antioxidant, Anti-hyperglycemic, Antihypertensive, &Antibacterial activity	(114)
		121°C for 20 min	Fenugreek, soybean, fava bean, & mung bean (sprouts or seedlings)	Autoclaving of sprouts (day2) and leaves (day 10)	Phenolic content, Antioxidant, Anti-hyperglycemic, & Antibacterial activity	(75)
		120°C for 10 min	Peruvian & Brazilian dry beans	Autoclaving of soaked beans	Antioxidant & Anti-hypertensive activity	(47)
		70 to 130°C for 1 to 5 min	Ginger, garlic, & turmeric	Thermal processing of spice powders on stovetop	Antioxidant and Anti-hyperglycemic activity	(115, 116)
		90°C for 3 or 5 h	Onion	Heat treatment of whole, crushed onions.	Anti-hyperglycemic activity	(117)
		100 to 180°C for 5 to 15 min	Sweet potato	Deep frying, boiling, steaming or baking	Phenolic content, Antioxidant, & Anti-hyperglycemic activity	(52)
		100°C for 30 to 90 min	Bian-Que Triple-Bean soup	Boiling of black soybean, mung bean, & adzuki bean	Anti-hyperglycemic activity.	(118)
		90 to 95°C for 5 to 120 min	Traditional Kenyan foods (cereals, legumes, oil seeds, & vegetables)	Blanching, soaking & cooking, or roasting	Phenolic content & Anti-hyperglycemic activity	(119)
		100 to 180°C for 5 to 15 min	Sweet potato	Deep frying, boiling, steaming or baking	Phenolic content, Antioxidant, &Anti-hyperglycemic activity	(52)
Chemical treatment	Calcium chloride		Fenugreek	Seed treatment followed by germination for 3 days	Antioxidant & Anti-hyperglycemic activity	(120)
Biological treatment	*R. oligosporus*	Active sporulating culture (3 weeks old)	Mung bean	Solid-state fermentation at room temperature for 20 days	Antioxidant, Anti-hyperglycemic, & Antibacterial activity	(121)
		Active sporulating culture (3 weeks old)	Fenugreek	Solid-state fermentation at room temperature for 20 days	Parkinson’s disease relevant L-DOPA^a^ content, Anti-hyperglycemic & Antibacterial activity	(122)
	Marine peptide hydrolysate	1mL/L	Black bean	Seed treatment at room temperature for 4 h	Antioxidant activity	(45)
Physical & chemical treatment	UV radiation & citric acid	253.7 nm (UV-C) & 0.5% (citric acid)	Apple	Fresh cut apples dipped in citric acid followed by exposure to UV-C	Phenolic content & Antibacterial activity	(123)
Physical & biological treatment	Dark germination & treatment with COS & marine peptide hydrolysate	1 to 10 g/L	Barley sprouts	Seed treatment followed by germination in dark for 6 days	Antioxidant &Anti-hyperglycemic activity	(72)
	Dark germination & treatment with fish protein hydrolysate &oregano extract	2 ml/L (fish protein hydrolysate) & 5 ml/L (oregano extract)	Velvet bean (*Mucuna pruriens*)	Seed treatment followed by germination in dark for 5 days	Phenolic content, antioxidant activity & Parkinson’s diseaserelevant L-DOPA content	(124)
Food synergies	Blueberry juice		Apple cider	Apple cider with 10% to 50% blueberry juice	Phenolic content, antioxidant, Anti-hyperglycemic, & Anti-hypertensive activity.	(125)
	Strawberry, blueberry, & peach.		Dairy & soy yoghurt	Commercially available yoghurt	Phenolic content, Antioxidant, Anti-hyperglycemic, & Anti-hypertensive activity	(126)
	Garlic, dill, onion, sweet red pepper, &cranberry		Cheese	Commercially available fruit enriched Roquefort, cheddar, feta, & English hard cheese	Anti-hyperglycemic & Anti-hypertensive activity	(127)
	*Rhodiola*		Kefir soymilk	Soymilk with 10% *Rhodiola*	Anti-hyperglycemic & Anti-hypertensive activity	(128)
	COS & vitamin C		Oregano & *Rhodiola*	Oregano or *Rhodiola* with 25% to 75% COS or vitamin C	Antioxidant, Anti-hyperglycemic, & Anti-hypertensive activity	(64)
	Pear		Blackberry	Blackberry with 25% pear	Antioxidant, Anti-hyperglycemic, & antihypertensive activity	(129)
	Sea buckthorn		Pear, apricot, peach, orange, green grape, apple, celery root, carrot, & parsley root	Fruit & vegetable smoothies with 25% or 50% sea buckthorn	Phenolic content, antioxidant, Anti-hyperglycemic, & Anticholinesterase activity	(130)
Fermentation with lactic acid bacteria (LAB)	Kefir culture-*L. lactis, L. brevis*, *Leuconostoc, & S. cerevisiae*	Commercially available culture	Soymilk	Fermentation at 25°C for 15 h	Isoflavone and Phenolic Content	(131)
	*L. acidophilus*	10% (v/v) inoculum	Cherry juice	Fermentation at 37°C for 72 h	Antioxidant, Anti-hyperglycemic, & Antibacterial activity	(55)
		10% (v/v) inoculum	Apple juice	Fermentation at 37°C for 72 h	Anti-hyperglycemic, Anti-hypertensive, & Antibacterial activity	(132)
		10% (v/v) inoculum	Pear juice	Fermentation at 37°C for 72 h	Anti-hyperglycemic activity	(133)
	*L. acidophilus, L. casei*, & *L. plantarum*	9.18 Log CFU/mL	Cashew apple juice	Fermentation at 30°C for 48 h	Phenolic & vitamin C content.	(134)
	*B. longum, L. casei, & L. plantarum.*	9.18 Log CFU/mL	Cashew apple juice	Fermentation at 37°C for 72 h	Antioxidant & Anti-hyperglycemic activity	(135)
	*L. acidophilus, L. bulgaricus, L. casei, S. thermophilus, L. rhamnosus, B. infantis*, & *B. longum & B. bifidum*	Commercially available culture.	Dairy yoghurt with 10% *Azadirachta indica*	Fermentation at 41°Cfor 6 h	Phenolic content, antioxidant, Anti-hyperglycemic, & Anti-hypertensive activity	(136)
	*L. acidophilus, L. bulgaricus, B. bifidum, L. casei, & S. thermophilus.*	Commercially available culture	Dairy yoghurt with cumin or coriander seeds (5 to 20g/100mL).	Fermentation at 41°C for 4 h	Phenolic content & Antioxidant activity	(137)
	*L. plantarum, Weissella cibaria, & Leuconostoc pseudomesenteroides*	6 Log CFU/mL	Papaya puree	Fermentation at 37°C for 48 h	Phenolic content, Antioxidant, & Anti-hyperglycemic activity	(138)
	*L. helveticus* & *B. longum*	10% (v/v) inoculum	Blackberry juice with 30% pear juice	Fermentation at 37°C for 48 h	Anti-hyperglycemic & Anti-hypertensive activity	(129)
	*L. plantarum, L. acidophilus, L. fermentum, L. paracasei*, & *L. rhamnosus.*	3% (v/v) inoculum	Apple juice	Fermentation at 37°C for 24 h	Anti-hyperglycemic activity	(139)
	*L. plantarum* & *L. helveticus.*	6.30 Log CFU/mL & 5.82 Log CFU/mL	Soymilk with Camu-camu (0% to 1%)	Fermentation at 37°C for 72 h	Anti-hyperglycemic & Anti-hypertensive activity	(57)
	*L. plantarum*	10% (v/v) inoculum	Sweet potato	Fermentation at 37°C for 72 h	Phenolic content, antioxidant, & Anti-hyperglycemic activity.	(140)
	*L. plantarum*	0.01% (w/w) inoculum	Bitter melon (*Momordica charantia*)	Fermentation at 37°C for 48 h	Antioxidant, Anti-hyperglycemic activity, & Beneficial Modulation of Gut Microbiota	(141, 142)

a levo-dihydroxy phenylalanine.

In a study with different rye varieties, high ferulic and benzoic acid content, as well as improved α-amylase enzyme inhibitory activity were observed in organically grown rye, while conventional production practices resulted in higher phenolic-linked antioxidant activity (42). In another study, higher catechin content was found in organic oat groats and rolled oat, when compared to the sample from conventional production practices (43). In a recent single crop-year study, higher phenolic bioactive-linked antioxidant and anti-hyperglycemic (α-amylase and α-glucosidase enzyme inhibitory activities) functional properties were observed in blackberry varieties with organic weed management and fertilization practices (mulching with feather meal) (97).

Oliveira et al. (146) reported high phenolic content, vitamin C, and soluble sugar content in organic tomato and indicated that the improvement was due to abiotic stress during fruit development stage under organic production practices. Therefore, effect of micro and macro environments both in soil rhizosphere and plant phyllosphere, which significantly varies between different crop years and due to different geographic regions might have greater influence on stress inducible and bioactive phenolic content which in turn dictates the health relevant nutritional qualities of plant foods. Significant effect of different crop-year and growing locations on phenolic-linked antioxidant and anti-hyperglycemic properties of rabbit-eye blueberry varieties were previously observed (59). Similarly, different crop years (environmental variations) resulted in significant variations in phenolic antioxidant content and altered anti-hyperglycemic functional qualities in serviceberry genotypes and oats in two different studies (43, 97). Furthermore, significant differences in phenolic linked antioxidant and anti-hyperglycemic functional qualities were observed in Tendergreen snap bean genotype between field and greenhouse growing conditions (46).

Impact of seasonal variations on phenolic-linked antioxidant, anti-diabetic and anti-hypertensive properties were also observed in leaf extracts of different Ginkgo trees (103). In another study, higher antioxidant activity was observed in extracts of *Swertia chirayita* grown in the wild, while high α-glucosidase enzyme inhibitory activity was found in extracts of cultivated plants (147). Therefore, results of all these studies indicated that growing environment and cultivation practices significantly influence phenolic bioactive content and optimum health protective functional qualities of plant foods. However, such variations also provide a unique opportunity to modulate and stimulate stress-inducible phenolic bioactives and to improve their functional benefits. Novel pre-harvest strategies targeting critical metabolic pathway regulation involved in the biosynthesis of stress-inducible phenolic bioactives can be advanced to enhance health-targeted functional qualities including glycemic control benefits in plant foods.

### Pre-Harvest Bio-Elicitation Strategy

An important metabolically modulating pre-harvest strategy is bio-elicitation with natural stress mimicking compounds to divert carbon flux towards the protective endogenous defense pathways in plants and concurrently stimulating stress inducible production of phenolic metabolites. Such stimulation of phenolic metabolite biosynthesis at critical reproductive and maturity stages can be harnessed to improve overall bioactive and nutritional qualities including chronic disease preventive functional properties in plant foods. In such stress response modulation harnessing strategies, non-toxic and water-soluble elicitors can be targeted as exogenous application or the plant can be exposed to controlled (in moderation) wounding, heat and cold shock, irradiation, UV or ozone treatments to improve stress inducible phenolic bioactive contents in plants (28).

In a field-based study, improvement of phenolic content, antioxidant activity, and anti-hyperglycemic property relevant *in vitro* α-amylase enzyme inhibitory activity were observed in select black bean genotypes with foliar application of water-soluble chitosan oligosaccharide during flowering stage (45). Foliar application of chitosan oligosaccharide 2 weeks before flowering also resulted into improvement in phenolic and flavonoid content in Greek oregano (*Origanum vulgare*) (110). Similarly, improvement in fruit quality and enhanced phenolic-linked antioxidant activity were observed in strawberry with pre-harvest application of water-soluble chitosan (111).

Application of short-term mild abiotic stress have also shown improvement of phenolic content and associated human health relevant functional qualities in food plants, especially in high-value specialty fruits. Manduri et al. (106) reported improvement in phenolic-linked antioxidant and anti-hyperglycemic properties in red wine derived from instantaneous heat-shock treated grapes (Pinot Noir). Similarly, enhanced phenolic content and higher anti-hyperglycemic properties (*in vitro* α-amylase and α-glucosidase enzyme inhibitory activities) were observed in white and red wine grape varieties following foliar application of ozone during fruit maturity stage (108). Though not many studies have investigated mild stress-induced improvement in human health relevant glycemic control protective functional properties of plant foods, stimulation of phenolic biosynthesis pathways and enhanced phenolic antioxidant content were observed with natural elicitor, ozone, heat and cold shock, salt stress, wounding, and UV-radiation treatments (107, 109, 112, 123) (**Table 2A** and **Figure 2**). Therefore, it is important to have field-based, multi-location, and multi-year studies in the future to better understand the efficacy of stress-modulating pre-harvest strategies for enhancing phenolic linked human health protective functional qualities especially targeting and harnessing wider T2D benefits.

## Post-Harvest Strategies to Improve Phenolic Bioactive-Linked Antioxidant and Anti-Diabetic Functionalities of Plant-Derived Foods and Beverages

**Figure 3:**
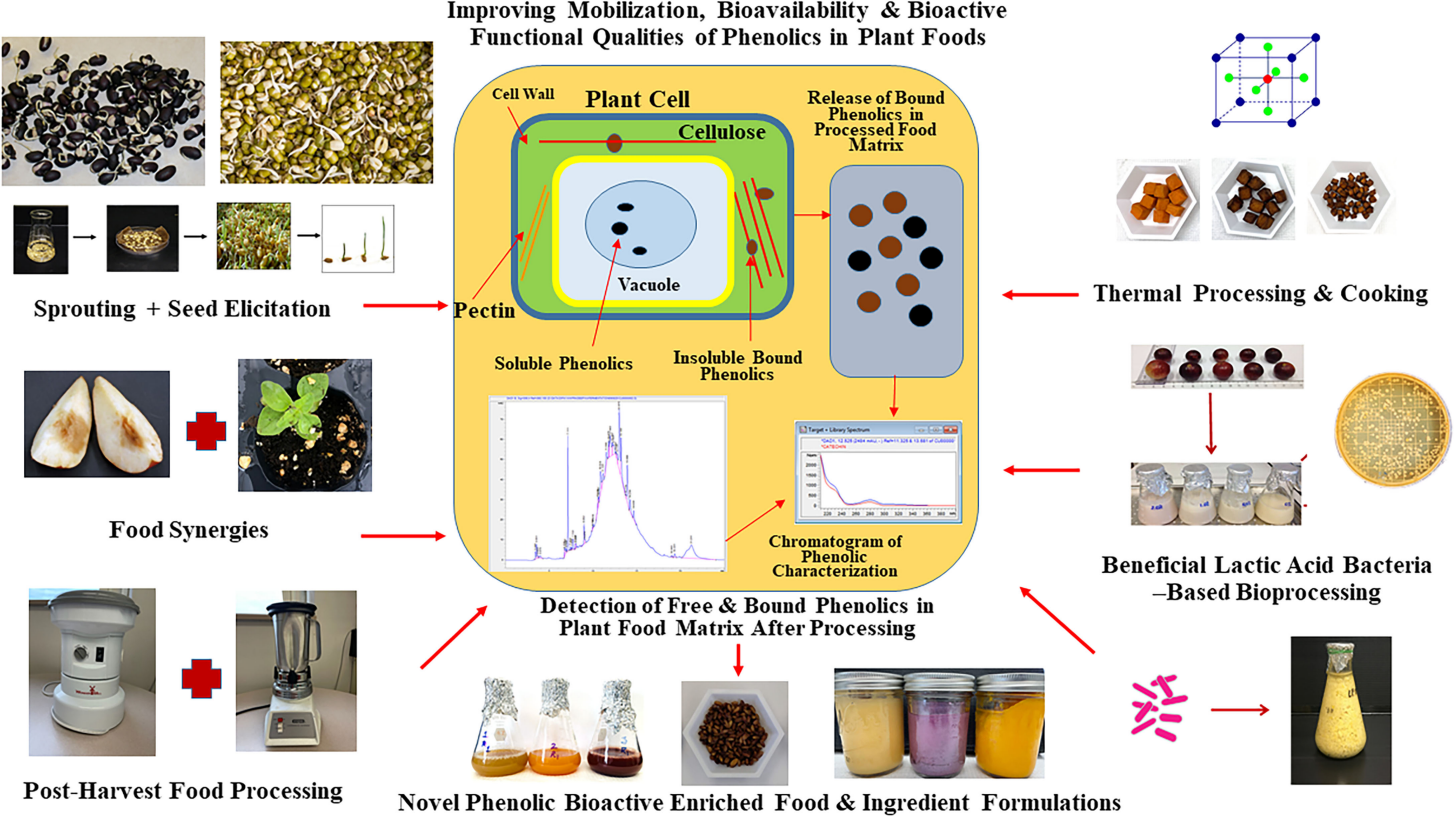
Post-harvest Bioprocessing Strategies to Enhance Mobilization, Bioavailability, and Bioactive Functionalities of Phenolics in Plant Foods. Sprouting, bio-elicitation, beneficial lactic acid bacteria-based fermentation, and different food processing and cooking strategies can be optimized and targeted to improve phenolic bioactive-linked antioxidant, anti-hyperglycemic and digestive health relevant benefits in plant foods from different geographical and ecological origins.

Globally, there is a growing interest in plant-based functional foods, food ingredients, and nutraceuticals targeting wider human health benefits and specifically to counter NCD-linked public health disparities. With modern scientific innovation and improved understanding on plant metabolites and their mobilization and bioavailability in plant food matrix, many strategies have evolved to enhance bioactive profiles and associated human health promoting functional qualities in foods and beverages derived from different plant sources (23, 148) (**Figure 3** and **Table 2B**). With increasing consumer awareness about health benefits of plant bioactives and dietary antioxidants, food industries are also investing in novel technologies to improve antioxidant and other bioactive-linked functional properties in plant-based processed foods. In this review, novel post-harvest approaches, which are safe, inexpensive, simple, and potentially effective strategies to improve phenolic linked anti-diabetic, anti-hypertensive, and human gut health benefits in plant foods and beverages are highlighted (**Table 2B**).

### Sprouting, Bioconversion and Biological Elicitation of Seeds

Sprouting is a traditional and inexpensive processing method that helps to biologically activate seeds upon the imbibition of moisture and trigger subsequent physiological and biochemical modification of seed biochemical components. During sprouting, enzymatic hydrolysis converts complex nutrient reserve of seeds into simple sugars and amino acids and such mobilization and solubilization of macronutrients also influence synthesis, mobilization, and bioavailability of free and bound bioactive compounds like phenolics. When integrated with novel seed elicitation approach, the bioavailability, and functional qualities of bioactives in sprouted seeds can be further enhanced. Seed elicitation with natural elicitors in combination with biological activation during sprouting can upregulate plant defense response pathways like anabolic pentose phosphate, shikimic acid, and phenylpropanoid pathways, which in turn improves biosynthesis of stress inducible phenolic metabolites and their bioactivity (**Figure 2**) (23, 27, 28).

In a recent study, improvement of *in vitro* α-glucosidase enzyme inhibitory activity and enhanced gallic acid content were observed in dark germinated (6 day) malting barley sprouts (72). Furthermore, improvement in phenolic-linked antioxidant and type 2 diabetes relevant anti-hyperglycemic properties were also found in dark germinated barley sprouts following seed elicitation with natural bioprocessed elicitors (chitosan oligosaccharide and fish protein hydrolysate) (72). Such an improvement in phenolic-linked health protective functional qualities in bio-elicited and sprouted barley was through upregulation of defense response pathways such as anabolic pentose phosphate pathway (73). Similar trend in improvement of phenolic –linked antioxidant functionalities through upregulation of anabolic pentose phosphate pathway was observed in bio-elicited (chitosan oligosaccharide and fish protein hydrolysate) and dark germinated black bean sprouts (45).

Previously, bioconversion of mung bean with *Rhizopus oligosporus* exhibited higher phenolic –linked antioxidant, α-amylase enzyme inhibition and anti-bacterial activity against ulcer causing bacteria *Helicobacter pylori* (121). Similar improvement in α-amylase enzyme inhibitory activity and anti-bacterial activity against *H. pylori* was found in *Rhizopus oligosporus* bioprocessed fenugreek (*Trigonella foenum-graecum*) extracts (122). Enhanced antioxidant and anti-diabetic functional qualities (*in vitro* α-amylase and α-glucosidase enzyme inhibition) was observed in sprouted (3 day) and bio-elicited (seed treatment with calcium chloride) fenugreek (120). In another study, improvement of phenolic-linked antioxidant activity was observed in dark germinated and bio-elicited *Mucuna pruriens* sprouts, while optimum anti-hyperglycemic relevant α-amylase and α-glucosidase enzyme inhibitory activity was found in cotyledon prior to the sprouting and bio-elicitation (124).

In a recent rat model-based study, improvement of glycemic control through reduced blood glucose level and hepato-protective properties were observed with treatment of methanolic extracts of broccoli (*Brassica cretica*) sprouts (149). Improved anti-hyperglycemic and anti-hyperlipidemic properties relevant for anti-diabetic benefits were also observed in rat model with sprouted quinoa-based yogurt (150). All these studies indicated that sprouting alone or in combination with bio-elicitation and food grade bioconversion are novel and effective approaches to enhance phenolic bioactive-linked antioxidant, anti-diabetic, and lipid metabolism benefits of plant foods. Such strategies can be targeted towards design functional food ingredients and nutraceuticals for wider T2D benefits.

### Thermal Processing and Cooking Optimization

Like sprouting, health-targeted optimization of cooking methods and food processing strategies can provide inexpensive and simple tools to the consumers for improving overall nutritional qualities of diverse foods. Such an optimization strategy can specifically target improved bioavailability and stability of bioactive compounds and their health protective functional qualities like glycemic control relevant benefits in cooked and processed foods. One of the most common and simple method is to use thermal treatments for mobilizing bioactive compounds and improving their functional properties in nutritionally relevant food matrix.

Previously, improvement of phenolic content, antioxidant activity, α-amylase, α-glucosidase, and ACE enzyme inhibitory activities were observed in thermal processed (autoclaved for 20 min) buckwheat and oat sprouts (2 day dark germinated) and seedlings (10 day leaves light germinated) (114). Additionally, anti-bacterial activity against *H. pylori* was also found in thermal processed sprouts and seedling extracts (114). Similar improvement in anti-diabetic functional qualities and anti-bacterial activity against *H. pylori* were observed in thermal processed fava bean, mung bean, soybean, and fenugreek sprouts (dark germinated) and micro seedlings (light germinated) (75). In another study, improved antioxidant and ACE inhibitory activity were found in thermal processed Peruvian and Brazilian bean varieties (47). The improvement in human health protective antioxidant, anti-hyperglycemic, anti-hypertensive, and anti-microbial functional qualities in sprouts and beans following thermal processing might be due to oxidation and polymerization of phenolic bioactives and subsequent modification in their structure-function relationship. Previously, thermally treated (toasted in conventional stove-top) spices (ginger-*Zingiber officinale*, garlic-*Allium sativum*, and turmeric-*Curcuma longa*) showed high phenolic linked antioxidant and enhanced α-glucosidase enzyme inhibitory activity (116). In another study, Hester et al. (115) found induced antioxidant activity in Wistar male rat following feeding with thermally treated garlic, ginger, and turmeric. Thermally treated onion (*Allium cepa*) extracts also showed improvement in anti-hyperglycemic functional qualities by suppressing carbohydrate absorption *via* inhibition of intestinal sucrose (117).

In addition to thermal processing, it is also important to optimize cooking conditions and related variables based on the bioactive stability and associated health targeted functional qualities of cooked foods. Common cooking methods like boiling, steaming, baking, frying, and microwave cooking can help to release embedded bioactive compounds in plant food matrix and potentially improve health-protective functional qualities such as antioxidant, anti-hyperglycemic, anti-hypertensive, and gut health benefits. However, without proper optimization cooking can also lead to loss of essential functional and nutritional qualities of food through significant physical and chemical changes. In a recent study with orange–fleshed sweet potato, Chintha (52) found higher retention of phenolic bioactives and improvement of associated antioxidant and anti-hyperglycemic functional qualities under optimized (cooking time, size of sample, and cooking temperature) cooking conditions. Higher antioxidant and anti-diabetic property relevant functional qualities were observed in oven baked and steamed sweet potato, when compared to boiled and deep-fried samples (52). Previously, Song et al. (118) optimized boiling time (30 min) of Bian-Que Triple Bean soup based on its α-glycosidase inhibitory activity and antioxidant property. Similarly, minimal processing (soaking + cooking and roasting) resulted into higher retention of phenolic compounds and associated antioxidant, and anti-hyperglycemic *(in vitro* α-amylase and α-glucosidase enzyme inhibitory activity) functional qualities in traditional Kenyan foods (finger millet-*Eleusine coracana*, amaranth-*Amaranthus* spp., pigeonpea- *Cajanus cajan*, field bean- *Vicia faba*, groundnut-*Arachis hypogea*, pumpkin-*Cucurbita pepo*, and sunflower seeds-*Helianthus annuus*) (119). Therefore, these studies indicated that optimizing cooking and processing conditions is essential to retain and improve bioactive-linked health functional qualities of diverse plant food matrix. Such health-targeted cooking and food processing optimization can provide critical insights into optimum bioactive and nutritional qualities supporting human health benefits.

### Health-Targeted Food Synergies

Addition of food ingredients that are rich source of bioactives to improve overall food and nutritional qualities is part of traditional food practices across the globe. In recent times, advanced knowledge on bioactive and nutritional benefits of specific food ingredients and improvement in food processing technologies have further expanded the use of combining different food matrices and ingredients to design novel foods with improved food quality and keeping quality. Food industries are also advancing strategies to find compatible food synergies for wider nutritional and bioactive targeted health benefits. Previous studies have advanced novel food synergy strategy by incorporating bioactive enriched edible plants to improve phenolic bioactive-linked anti-diabetic functional qualities of different plant and dairy-based foods (125, 129).

In a study targeting cranberry-based herbal synergy, high *in vitro* α-glucosidase and α-amylase enzyme inhibitory activities were observed in cranberry: *Rhodiola rosea* (75:25) combination, while cranberry: rosemary (*Salvia rosmarinus*) (75:25) combination had high anti-hypertensive property relevant ACE inhibitory activity (151). In another study, improvement in phenolic bioactive mobilization and associated anti-hyperglycemic and anti-hypertensive functional qualities were observed when Kefir-culture mediated fermentation of soymilk was supplemented with *Rhodiola* (2%) extracts (128). Improved anti-hyperglycemic functional qualities of Kefir-culture mediated soymilk was positively correlated with tyrosol and salidroside content, two major health protective phenolic bioactive compounds of *Rhodiola* (128). Like fermented and *Rhodiola* supplemented soymilk, cranberry enrichment of cheese (Cheddar, feta, and Roquefort) also showed improvement in α-amylase and α-glucosidase enzyme inhibitory activity relevant for anti-hyperglycemic benefits (127). High anti-hyperglycemic and anti-hypertensive property were found in yogurt enriched with different plant extracts like neem (*Azadirachta indica*) (136), coriander, and cumin seeds (137). Integration of chitosan oligosaccharide resulted in enhanced antioxidant activity in medicinal herb extracts (oregano and *Rhodiola*), while vitamin C and herb combinations exhibited improved α-glucosidase and ACE inhibitory activity (64). Many recent reviews have highlighted the benefits of integrating bioactive enriched medicinal plants and herbs for improving anti-diabetic and anti-hypertensive relevant functional properties of foods and for therapeutic applications (152, 153).

Berries that are a rich source of dietary and water-soluble antioxidants are also excellent targets for novel food synergies, specifically to improve anti-inflammatory, anti-hyperglycemic, and anti-dyslipidemic properties of foods and beverages. Addition of blueberry juice (20%) in apple cider (80%) showed improvement in phenolic-linked antioxidant and *in vitro* α-glucosidase enzyme inhibitory activity (125). Similarly, higher antioxidant, anti-hyperglycemic, and anti-hypertensive properties were observed in 30:70 blackberry: pear combination (129, 154). In a recent study, enrichment with sea buckthorn berry (*Elaeagnus rhamnoides* L.) resulted in higher anti-cholinesterase, anti-hyperglycemic, and antioxidant properties in fruit (75%) and vegetable (25%) combined smoothies (130). Such integration of compatible fruit extracts with complementary bioactive profile is an effective approach to enhance human health protective functional qualities of minimally processed fruit-based foods and beverages. In addition to compatible fruits, phenolic enriched dried berry extracts can also be added to other plant-based food matrices to improve wider health benefits. Significant reduction of glucose (24-74%) production was observed in rat hepatocytes, when blueberry phenolic enriched defatted soybean flour was incorporated in very high fat diet formulations (155). Similarly, addition of Saskatoon (serviceberry-*Amelanchier* spp.) berry powder (5%) in regular mouse diet resulted into reduction of blood and urine glucose level in diabetic mouse model (156). A recent meta-analysis-based study indicated that consumption of phenolic enriched foods alone or in combination with anti-diabetic drugs has significant potential to lower blood glucose levels in individuals with T2D or at pre-diabetic state (157). Therefore, health targeted food synergy by combining phenolic enriched food matrices is an effective dietary and therapeutic approach to counter breakdown of glucose metabolism and associated health risks for prevention and management of type 2 diabetes.

### Bioprocessing Using Lactic Acid Bacteria (LAB)-Based Fermentation

Fermentation of plant and dairy-based food substrates with beneficial and edible microorganisms is a widely popular traditional food practice, which can be found in all cultures and geographic regions across the globe. There is a renewed interest in fermented foods due to the growing perception about its wider health benefits, specifically with more informed understanding about the role of improved gut health in reducing risks of chronic diseases (NCDs) (158). In the context of improved food quality through benefits of fermentation helps to enhance mobilization and bioavailability of bioactive metabolites in fermented end-products and thus increase overall nutritional and health protective functional properties. Improved isoflavone and phenolic acid content was reported in Kefir-fermented soymilk (131). Advancement in food technologies has also supported optimization of more controlled fermentation both for small domestic and pilot scale (food industries) to improve specific health targeted benefits like redox and glycemic control protective properties in fermented foods and beverages. Among beneficial microorganisms, probiotic relevant lactic acid bacteria (LAB) based fermentation is most widely used bioprocessing tool to improve nutritional and keeping quality of dairy and plant-based foods (148).

Many previous studies have found improvement in glucose metabolism, anti-hypertensive, antioxidant, anti-dyslipidemia, and vascular health protective functional properties in dairy and plant-based food substrates following controlled fermentation with LAB (55, 132–134, 159, 160). Cohort study based meta-analysis also revealed reduced risk of T2D with higher intake of yogurt, the most popular and traditional LAB fermented food (161, 162). In addition to improvement in mobilization of phenolics that influence health relevant functional qualities of fermented dairy foods, release of other bioactive compounds like peptides in fermented end products also enhance glycemic control and anti-hypertensive property relevant functional benefits (163, 164).

Like the results of dairy-based food substrates, LAB-based fermentation is also an effective dietary strategy to improve health protective bioactive functional qualities such as anti-hyperglycemic and anti-hypertensive properties of diverse plant-based food substrates (148). However, the improvement of bioavailability and bio-accessibility of bioactive compounds of fermented plant food largely depends on type and source of plant substrate, type of LAB strains, and total fermentation time, and methods of bioprocessing. A study with *in vitro* digestion of papaya (*Carica papaya*) puree revealed higher recovery of individual phenolic compounds like chlorogenic, vanillic, syringic, ellagic, ferulic acids, catechin, epicatechin, and quercetin following fermentation with *Lactoplantibacillus plantarum* (L75*D7) and *Weiissella cibaria64* (W64*D7) strains (138). Improvement in antioxidant and anti-hyperglycemic functional qualities were observed with LAB-based fermentation of aqueous extracts of cherry (*Prunus avium*), pear, and apple (55, 132, 133). Similarly, improved anti-hyperglycemic and anti-hypertensive property were observed in pear: blackberry (70:30) fruit synergy following fermentation with beneficial LAB (*L. helveticus*, and *Bifidobacterium longum*) (129). Significant improvement in α-glucosidase enzyme inhibitory activity was found with LAB fermentation of Aksu apple juice (139).

In addition to common fruits and berries, LAB fermentation is particularly relevant in improving bioactive functional qualities and to remove anti-nutrients of underutilized fruits and plant food byproducts. Enhanced *in vitro* α-glucosidase, α-amylase, and moderate ACE enzyme inhibitory activities were observed in camu-camu and soymilk combination after fermenting with *L. plantarum* and *L. helveticus* (57). Similarly, improvement in antioxidant and anti-hyperglycemic functional properties were also observed in LAB fermented cashew (*Anacardium occidentale*) apple juice (135). Improvement in phenolic bioactive-linked antioxidant, and anti-hyperglycemic functional qualities were also observed in different flesh-colored sweet potato following *L. plantarum* based fermentation (140). In recent rat model-based studies, *Momordica charantia* extracts fermented with *L. plantarum* showed improvement in lipid metabolism, glycemic control, and beneficial alteration in gut microbiota composition relevant for wider T2D benefits (141, 142). Therefore, LAB based fermentation can be rationally recruited as a novel bioprocessing tool to improve bioactive functional qualities like anti-diabetic, anti-dyslipidemia, and cardiovascular health protective properties in dairy and plant-based food substrates. Such fermentation based bioprocessing strategy are also relevant to develop functional food ingredients and nutraceuticals targeting T2D benefits. Additionally, due to wider use of fermentation in traditional food practices, this bioprocessing approach is also pertinent in developing culturally relevant foods with bioactive enriched health benefits.

## Conclusion

Narrow dietary choices high in macronutrients with higher intake of refined and hyper-processed soluble carbohydrate rich foods, unhealthy lifestyles like physical inactivity, and genetic pre-disposition are major contributing factors in increasing prevalence of T2D and related mortality worldwide. To counter and reduce the risks of common T2D and its co-morbidity, it is important to improve our daily diet by widening plant food diversity with balanced and superior bioactive and wider nutritional quality rich profiles. Dietary preventive strategies to counter T2D at early stages of disease development is specifically important for addressing growing health care costs and economic burden. Among health protective and health promoting bioactives, stress-inducible phenolic bioactives of plant foods are drawing wider attention in advancing healthy dietary strategies based on their diverse functional properties like antioxidant, anti-hyperglycemic, anti-hypertensive, anti-dyslipidemic, and gut health benefits. However, optimization of wide variability and diversity in content and composition of phenolic bioactives among plant food sources is essential for delivery consistency and therefore is a major challenge for effective integration in dietary and therapeutic strategies to deliver optimum functional health benefits. Therefore, it is necessary to screen and optimize phenolic bioactive content, composition, and functional qualities of plant food sources across farm to fork food chain to incorporate them in health targeted food applications. Advancements in metabolically relevant screening strategies and growing empirical evidence on specific health protective role of phenolic bioactives of plant foods against chronic diseases like T2D have widened the scope of finding suitable plant food and food ingredients with optimum bioactive profile and health relevant functional qualities for delivery. Additionally, novel pre-harvest and post-harvest strategies based on improved understanding of biosynthesis, mobilization, bioavailability, and bioaccessibility of stress inducible phenolics have enabled to further enhance bioactive content and health promoting nutritional qualities in wide arrays of plant foods. Studies highlighted in this review clearly indicate that phenolic bioactive enriched plant foods are safe and inexpensive dietary targets to counter common health risks associated with T2D. However, extensive clinical and epidemiological studies are required to improve our understanding on glycemic control protective mechanisms of plant phenolics relevant for wider dietary and therapeutic solutions against T2D. Achieving health targeted dietary benefits with bioactive enriched plant food diversity in diverse global ecologies are extremely important to address wider T2D-linked public health disparities, and specifically to improve nutrition, health, and well-being of marginalized and less developed health care communities globally.

## Author Contributions

DS and KS: Conceptualization. DS and AC: Review literature collection. DS, AC, and KS: writing—original draft. DS and AC: Creation of figures and tables. KS and DS: Review and Editing. All authors contributed to the article and approved the submitted version.

## Conflict of Interest

The authors declare that the research was conducted in the absence of any commercial or financial relationships that could be construed as a potential conflict of interest.

## Publisher’s Note

All claims expressed in this article are solely those of the authors and do not necessarily represent those of their affiliated organizations, or those of the publisher, the editors and the reviewers. Any product that may be evaluated in this article, or claim that may be made by its manufacturer, is not guaranteed or endorsed by the publisher.
